# Magnitude and Factors Associated With Short Birth Intervals Among Reproductive‐Age Women in Awi Zone, Northwest Ethiopia: A Community‐Based Cross‐Sectional Study

**DOI:** 10.1002/hsr2.72440

**Published:** 2026-04-22

**Authors:** Nigussie Adam Birhan, Kefale Tilahun Getahun, Zelalem Meraf Wolde, Kassahun Animut Metkie, Denekew Bitew Belay, Yegnanew A. Shiferaw

**Affiliations:** ^1^ Department of Statistics, College of Natural and Computational Science Injibara University Injibara Ethiopia; ^2^ Department of Statistics, College of Natural and Computational Science Dilla University Dilla Ethiopia; ^3^ Department of Statistics, College of Science Bahir Dar University Bahir Dar Ethiopia; ^4^ School of Health Systems and Public Health, Faculty of Health Sciences University of Pretoria Pretoria South Africa; ^5^ Department of Statistics University of Johannesburg Johannesburg South Africa

**Keywords:** Awi zone, binary logistic regression model, birth spacing, reproductive age women, short birth interval

## Abstract

**Background and Aims:**

Short birth interval is defined as an interval of less than 33 months between two consecutive live births. It affects maternal, perinatal, neonatal outcomes, and infant development. Thus, this study aimed to identify the associated factors of short birth interval among reproductive‐age women in the Awi zone.

**Methods:**

A community‐based cross‐sectional study was conducted among 1144 reproductive‐aged women from February to June 2023 using a multistage sampling technique. Data were collected using structured and pretested questionnaires. Data were entered into EpiData version 4.0.2 and exported to STATA 18 for further analysis. Bivariable and multivariable logistic regression models were fitted. In the multivariable logistic regression, adjusted odds ratios with 95% confidence intervals were used to identify factors significantly associated with short birth intervals.

**Results:**

The prevalence of short birth interval was 29.20% (95% CI: 26.63, 31.90%). Primary educated hasband/parent [AOR = 0.54; 95% CI: 0.32, 0.91; *p* = 0.021], use of contraceptive [AOR = 0.04; 95% CI:0.02, 0.06; *p* < 0.001], rural resident [AOR = 2.03; 95% CI:1.10, 3.76; *p* = 0.023], rich wealth index [AOR = 0.44; 95% CI: 0.23, 0.86; *p* = 0.016], above 18 years old at first marriage [AOR = 0.61; 95% CI: 0.40, 0.94; *p* = 0.025], greater than 24 months breastfeeding [AOR = 0.46; 95% CI: 0.30, 0.71; *p* < 0.001], and six and more ideal number of children [AOR = 2.05; 95% CI: 1.30, 3.22; *p* = 0.002] were associated factors of short birth interval.

**Conclusion:**

In the Awi zone, the prevalence of short birth interval is still high. Therefore, policy makers and stakeholders strengthening family planning services, promoting optimal breastfeeding practices, delaying early marriage, improving women's education, and socioeconomic status are essential to encourage optimum birth interval. Furthermore, targeted community‐based interventions, particularly in rural areas, are recommended to reduce short birth intervals.

**Abbreviations**
AOR: adjusted odds ratio
CI: confidence interval
COR: crude odds ratio
FMoH: Federal Ministry of Health
IVF: variance inflation factor
WHO: World Health Organization

## 1 Background

Birth interval refers to the time period between two consecutive live births and major predictor of fertility patterns globally [1, 2]. Short birth interval is defined as an interval of less than 33 months between two consecutive live births [3]. It is a global public health problem that affects mothers, neonatal outcomes, and child well‐being [4]. Closely spaced pregnancies contribute to malnutrition, maternal depletion syndrome, milk diminution, and competition for care and other resources among siblings [5]. These conditions have been associated with numerous serious outcomes for neonates with low birth weight, stillbirth, newborn and child mortality, maternal mortality, adverse effects on intellectual ability, physical growth and development, premature rupturing of membranes, preterm birth, uterine and placental bleeding, and gestational diabetes [4, 6, 7]. Optimum birth interval has health benefits for both mothers and children in minimizing births, risks due to premature abortions, and unintended pregnancies. It further promotes infant growth by enhancing the nutritional condition of the preceding child and has the greatest economic, physical, and social value for the household and community [3]. Fertility is the main component of population growth and plays a major role in changing the size and structure of a given population in the world [8, 9]. Over recent decades, the global population has grown quickly, with the growth rate remaining higher in developing countries than in developed countries [10]. In 2023, the total fertility rate was 2.3 births per woman in the world [11], whereas in sub‐ Saharan Africa (SSA), the rate is considerably higher at 4.3 [12].

Fertility, maternal, and child mortality were highest in Sub‐Saharan Africa, like Ethiopia. Consequently, understanding the level of short birth interval and factors affecting short birth interval is critical for nations to develop population policies aimed at decreasing fertility rates [13].

In 2023, Ethiopia is the second most populous country in Africa, next to Nigeria, with a higher fertility rate of 4.0 [14]. High fertility rate limits women's potential participation in economic development, making it difficult to be productive members of society, and it can influence the total economic, political, and social facets of a given nation [15]. In addition to health ramifications, the short birth interval threatens the country's growth efforts. It restricts the future involvement of women in economic growth, making it impossible for them to be active members of society. Moreover, when a newborn baby comes, the family will invest more resources to care for the newborn, although the other children will be left behind a scarce portion of the resources [16].

In the last decades, Ethiopia has been planning to reduce the high fertility rate and has been showing a decline, but the fertility rate is still high due to different factors [10, 16]. Empirical evidence from many different settings has identified several factors of short birth interval including breast feeding, contraceptive use, maternal education [2, 17], household wealth status [18], residence [19], breastfeeding duration [20], parity [21], maternal age [22], media exposure [22], and age at first marriage [23]. However, studies assessing short birth intervals in the Awi Zone are limited, with only a few regional studies available from Amhara and neighboring regions [15, 24], and no comprehensive investigation has been conducted in the Awi zone. Thus, the aim of this study was to identify the factors associated with short birth interval among reproductive‐age women in the Awi zone. It is hoped that the findings of the study will help to enhance policymakers' awareness of effective and efficient planning and distribution of resources to improve the birth rate among women of reproductive age in the Awi zone.

## 2 Methods and Materials

### 2.1 Study Design, Setting, and Period

A community‐based cross‐sectional study was carried out in the Awi Zone of the Amhara Region, northwestern Ethiopia, from February to June 2023. It is located 114 km from Bahir Dar, the capital of the Amhara region, and 449 km from Addis Ababa, which is the capital city of Ethiopia. Administratively, the zone consists of fifteen districts (six urban and nine rural).

### 2.2 Study and Source Population

The source of population included all reproductive‐age women in the Awi zone who gave birth within the 5 years preceding data collection. However, all reproductive‐age women residing in the selected kebele in Awi zone who gave birth within the 5 years prior to the data collection were the study population.

### 2.3 Inclusion and Exclusion Criteria

All women who gave birth within 5 years and have at least two consecutive live births were incorporated under this study. However, mothers who had history of preceding abortion between the last two consecutive births, had resided in the study area for less than 6 months, were critically ill or mentally ill women, or those who were not volunteers to participate in the study were excluded.

### 2.4 Sample Size Determination and Sampling Technique

Sample size was computed based on single population proportion formula by using: confidence level = 95%, margin of error (*d*) = 3%, and the prevalence of Short birth interval among women of childbearing age in northern Ethiopia (23.3%), which gave a relatively larger sample size, which was taken into consideration [4]. After taking into account the 10% non‐respondent rate and the fact that the sampling technique involves multiple stages of sampling, a design effect of 1.5 was taken into account, leading to a final sample size of 1202.

Participants were selected through multistage sampling approach, involving random selection of districts and kebeles, followed by systematic sampling of households using proportional to population size [25]. After excluding non‐respondents and missing 1144 women were included in the final analysis, yielding a response rate of 95.17%.

### 2.5 Data Collection Technique and Quality Control

Data were collected using a structured, pretested questionnaire translated into local languages and back‐translated for consistency. Trained health professionals conducted the interviews under supervision, and a 5% pretest ensured tool reliability and data quality.

### 2.6 Study Variable

In this study, the response variable was the short birth interval of women, which is defined as an interval less than 33 months between two successive live births and coded as 1 and intervals 33 and above months were defined as optimum birth interval and coded as 0, based on guidelines proposed in the WHO Technical Consultation on Birth Spacing and other studies [3]. The independent variable includes mother's educational level, mother's occupational status, current age of mother, household wealth index, husband/partner educational level, husband/partner occupational status, duration of breastfeeding status, child sex, mother's marital status, parity, mother's age at first marriage, place of delivery, distance to health facility, mother's religion, ideal number of children, media exposure, place of residence, and contraceptive use before last pregnancy. The household wealth index was constructed using principal component analysis (PCA) based on household assets (radio, television, and livestock), housing materials, water source, and sanitation facilities. The resulting PCA scores were used to rank households and categorize them into five quintiles (poorest, poorer, middle, richer, and richest). Further, these quintiles were recoded into three categories: poor (poorest and poorer), middle, and rich (richer and richest) [26].

### 2.7 Data Management and Analysis

The data were entered and cleaned in EpiData version 4.0.2 and exported to STATA 18 for further analysis. Frequency and percentages of the variables were presented using descriptive statistics. Bivariable and multivariable logistic regression model was used to assess the associations between dependent and independent variables. Bivariable logistic regression analysis, the crude odds ratio (COR) was carried out to identify associations between the short birth interval and each independent variable. All variables with *p*‐value < 0.25 in the bivariable analysis were chosen for the multivariable logistic regression analysis to compensate for confounders [27]. In the multivariable logistic regression analysis, the adjusted odds ratio (AOR) was used to determine factors associated with short birth interval among reproductive‐age women at a 95% confidence interval (CI). Significant predictors were defined as factors with a *p*‐value of less than 0.05. Multicollinearity among independent variables was assessed using the Variance Inflation Factor (VIF), and no significant multicollinearity was observed (VIF < 10). The goodness‐of‐fit of the final model was evaluated using the Hosmer–Lemeshow test, which indicated adequate model fit (*p* > 0.05).

## 3 Results

### 3.1 Socio‐Demographic Characteristics of the Study Participants

In this study, a total of 1144 reproductive‐age women were included with a response rate of 95.17%. Of the total respondents, the majority of mothers, 774 (67.66%), were living in rural areas. About 598 (52.27%) women were in the age group of 25–34 years. The majority of women, 1093 (95.54%), were married, 505 (44.14%) were from poor households, and 678 (59.27%) were unable to read and write (Table 1).

**Table 1:** Socio‐demographic characteristics of reproductive‐age women in Awi zone, Northwest Ethiopia, 2023.

Characteristics	Categories	Frequency	Percentage
Residence	Urban	370	32.34
	Rural	774	67.66
Sex of child	Male	636	55.59
	Female	508	44.41
Religion	Orthodox	1077	94.14
	Other	67	5.86
Husband/partner education	Illiterate	352	32.12
	Able to write and read	239	21.81
	Primary	287	26.19
	Secondary	81	7.39
	Higher	137	12.50
Marital status	Married	1093	95.54
	Other^a^	51	4.46
Husband/partner occupation	Farmer	778	70.92
	Government employed	84	7.66
	Merchant	128	11.67
	Other^b^	107	9.75
Mother education	No education	678	59.27
	Primary	256	22.38
	Secondary and above	210	18.36
Mother occupation	Farmer	701	61.28
	Government Employed	51	4.46
	Housewife	342	29.90
	Other^b^	50	4.37
Age of mother	Below 24	47	4.11
	25–34	598	52.27
	35 and above	499	43.62
Maternal age during first marriage	Less than 18	258	22.55
	18 and above	886	77.45
Wealth index	Poor	505	44.14
	Middle	227	19.84
	Rich	412	36.01
Media exposure	No	559	48.86
	Yes	585	51.14

^a^ Widowed/divorced/separated.

^b^ Daily labor/merchant.

### 3.2 Obstetric and Health Service‐Related Characteristics of Reproductive‐Age Women

Of the total 1144 reproductive‐age women, about 541 (47.29%) of the women reported 6 and more ideal numbers of children, including the current birth, and 496 (43.36%) had no use of contraceptives before the last pregnancy. Nearly 379 (33.13%) of women perceived distance to access health care as a big problem (Table 2).

**Table 2:** Obstetric and health service‐related characteristics of reproductive‐age women in Awi zone, Northwest Ethiopia, 2023.

Characteristics	Categories	Frequency	Percentage
Parity	2 and below	403	35.23
	3–4	409	35.75
	Above 4	332	29.02
Ideal number of children	Less than 6	603	52.71
	6 and above	541	47.29
Place of delivery	Home	77	6.73
	Health facility	1,067	93.27
Distance to health facility	Not big problem	765	66.87
	Big problem	379	33.13
Contraception usage	No	496	43.36
	Yes	648	56.64
Duration of breastfeeding	0–12 months	329	28.76
	13–23 months	328	28.67
	24 and above months	487	42.57

### 3.3 Prevalence of Short Birth Interval

**Figure 1:**
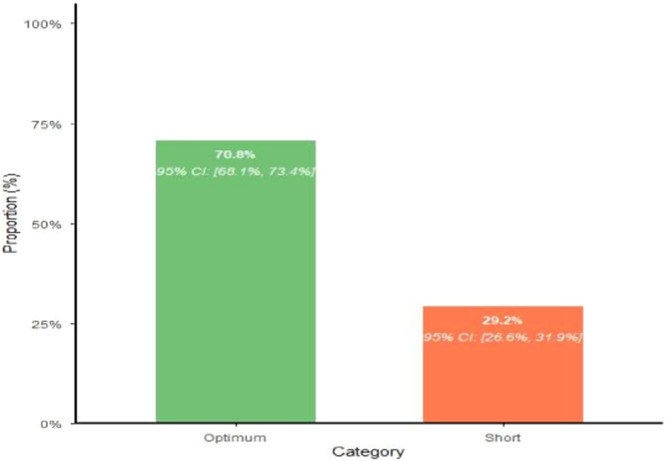
Prevalence of short birth interval among reproductive‐age women in Awi zone (29.2%, 95% CI: 26.6–31.9%; *n* = 334/1144).

The finding of this study revealed that the prevalence of short birth interval among reproductive‐age women in Awi zone was 29.20% (95% CI: 26.63, 31.90%) (Figure 1).

### 3.4 Factors Associated With Short Birth Interval

In this study, husband/partner education level, use of contraceptive before last pregnancy, place of residence, wealth index of household, mother age at first marriage, duration of breastfeeding, and ideal number of children were statistically associated with short birth interval at 95% confidence level. The Hosmer–Lemeshow goodness‐of‐fit test indicated adequate model fitness (*χ* ^2^ = 3.16, *p* = 0.206). The mean VIF was 3.52, indicating no multicollinearity. Women who resided in rural areas had 2.03‐fold higher odds of experiencing short birth intervals as compared to those from the urban areas (AOR = 2.03; 95% CI: 1.10, 3.76; *p* = 0.023). The odds of short birth interval among women aged 18 and above years at first marriage were 0.61 times lower compared with women whose age at their first marriage was below 18 years (AOR = 0.61; 95% CI: 0.40, 0.94; *p* = 0.025).

The odds of a short birth interval were 0.04 times lower among women who used a contraceptive before their last pregnancy compared to those who did not use contraceptive before their last pregnancy (AOR = 0.04; 95% CI: 0.02, 0.06; *p*< 0.001). Breastfeeding duration of the preceding child greater than or equal to 24 months decreased the risks of the short birth interval by 0.46, compared to breastfeeding duration of less than 12 months (AOR = 0.46; 95% CI: 0.30, 0.71; *p* < 0.001). Women who had a primary‐educated husband/partner had 0.54‐fold lower odds of short birth intervals compared to husband/partner with illiterate (AOR = 0.54; 95% CI: 0.32, 0.91; *p* = 0.021). Women from rich households had 0.44‐fold lower odds of experiencing short birth interval than women from poor households (AOR = 0.44; 95% CI: 0.29, 0.86; *p* = 0.016). Moreover, women who have a desire of six and more children had 2.05‐fold lower odds of short birth interval compared to those women having desire of less than six children (AOR = 2.05; 95% CI; 1.30, 3.22; *p* = 0.002) (Table 3).

**Table 3:** Bivariable and multivariable logistic regression analysis of short birth interval and associated risk factors in Awi Zone, Northwest Ethiopia (*n* = 1144).

Characteristics	Categories	Optimum (%)	Short (%)	COR (95% CI)	AOR (95% CI)	*p*‐value
Residence	Urban (Ref.)	298 (36.79)	72 (21.56)	1	1	
	Rural	512 (63.21)	262 (78.44)	2.12 (1.57, 2.85)	2.03 (1.10, 3.76)	0.023
Sex of child	Male (Ref.)	465 (57.41)	171 (51.20)	1	1	
	Female	345 (42.59)	163 (48.80)	1.29 (0.99, 1.66)	1.09 (0.76,1.56)	0.646
Religion	Orthodox (Ref.)	768 (94.81)	309 (92.51)	1	1	
	Other	42 (5.19)	25 (7.49)	1.48 (0.89, 2.47)	1.68 (0.72, 3.83)	0.722
Husband/partner education	Illiterate (Ref.)	223 (28.96)	129 (39.57)	1	1	
	Able to write	146 (18.96)	93 (28.53)	1.10 (0.9, 1.55)	0.96 (0.61, 1.53)	0.875
	Primary	230 (29.87)	57 (17.48)	0.43 (0.30, 0.62)	0.54 (0.32, 0.91)	0.021
	Secondary	64 (8.31)	17 (5.21)	0.46 (0.26, 0.82)	0.65 (0.28, 1.52)	0.323
	Higher	107 (13.90)	30 (9.20)	0.49 (0.31, 0.77)	1.56 (0.62, 3.95)	0.345
Marital status	Married (Ref.)	768 (94.81)	325 (97.31)	1	1	
	Other^a^	42 (5.19)	9 (2.69)	0.51 (0.24, 1.05)	2.30 (0.41, 12.84)	0.344
Husband/partner occupation	Government (Ref.)	69 (8.96)	15 (4.59)	1	1	
	Farmer	530 (68.83)	248 (75.84)	2.15 (1.21, 3.84)	0.60 (0.16, 2.28)	0.457
	Merchant	83 (10.78)	45 (13.76)	2.49 (1.28, 4.85)	1.91 (0.64, 5.73)	0.250
	Other^b^	88 (11.43)	19 (5.81)	0.99 (0.47,2.096)	0.68 (0.21, 2.21)	0.523
Mother education	No education (Ref.)	444 (54.81)	234 (70.06)	1	1	
	Primary	208 (25.68)	48 (14.37)	0.44 (0.31, 0.62)	0.90 (0.51, 1.59)	0.715
	Secondary and above	158 (19.51)	52 (15.57)	0.62 (0.44, 0.89)	1.54 (0.73, 3.23)	0.254
Mother occupation	Farmer (Ref.)	467 (57.65)	234 (70.06)	1	1	
	Government employed	42 (5.19)	9 (2.69)	0.43 (0.21, 0.89)	1.03 (0.27, 3.96)	0.907
	Housewife	264 (32.59)	78 (23.35)	0.59 (0.44, 0.79)	0.60 (0.31, 1.19)	0.145
	Other^b^	37 (4.57)	13 (3.89)	0.70 (0.37, 1.35)	0.68 (0.21, 2.22)	0.521
Age of mother	Below 24 (Ref.)	41 (5.06)	6 (1.80)	1	1	
	25–34	441 (54.44)	157 (47.01)	2.43 (1.01, 5.84)	1.66 (0.54, 5.13)	0.381
	35 and above	328 (40.49)	171 (51.20)	3.56 (1.48, 8.56)	0.98 (0.28, 3.38)	0.971
Parity	2 and below (Ref.)	337 (41.60)	66 (19.76)	1	1	
	3–4	295 (36.42)	114 (34.13)	1.97 (1.40, 2.78)	1.05 (0.60, 1.81)	0.876
	Above 4	178 (21.98)	154 (34.13)	4.42 (3.14, 6.21)	1.28 (0.62, 2.64)	0.509
Ideal number of children	Less than 6 (Ref.)	489 (60.37)	114 (34.13)	1	1	
	6 and above	321 (39.63)	220 (65.87)	2.94 (2.25, 3.84)	2.05 (1.30, 3.22)	0.002
Maternal age during first marriage	Less than 18 (Ref.)	149 (18.40)	109 (32.63)	1	1	
	18 and above	661 (81.60)	225 (67.37)	0.47 (0.35, 0.62)	0.61 (0.40, 0.94)	0.025
Wealth index	Poor (Ref.)	322 (39.75)	183 (54.79)	1	1	
	Middle	163 (20.12)	64 (19.16)	0.69 (0.49, 0.97)	0.73 (0.44, 1.22)	0.227
	Rich	325 (40.12)	87 (26.05)	0.47 (0.35, 0.64)	0.44 (0.23, 0.86)	0.016
Media exposure	No (Ref.)	382 (47.16)	177 (52.99)	1	1	
	Yes	428 (52.84)	157 (47.01)	0.79 (0.61, 1.02)	1.03 (0.66, 1.61)	0.890
Place of delivery	Home (Ref.)	53 (6.54)	24 (7.19)	1		
	Health facility	757 (93.46)	310 (92.81)	0.90 (0.55, 1.49)	Not included^c^	—
Distance to health facility	Not big problem (Ref.)	555 (68.52)	210 (62.87)	1	1	
	Big problem	255 (31.48)	124 (37.13)	1.29 (0.98, 1.68)	0.69 (0.46, 1.03)	0.068
Contraception usage	No (Ref.)	196 (24.20)	300 (89.82)	1	1	
	Yes	614 (75.80)	34 (10.18)	0.04 (0.03, 0.05)	0.04 (0.02, 0.06)	< 0.001
Duration of breastfeeding	0–12 months (Ref.)	213 (26.30)	116 (34.73)	1	1	
	13–23 months	228 (28.15)	100 (29.94)	0.81 (0.58, 1.12)	0.76 (0.47, 0.22)	0.252
	24 and above months	369 (45.56)	118 (35.33)	0.59 (0.43, 0.80)	0.46 (0.30, 0.71)	< 0.001

^a^ Widowed/divorced/separated.

^b^ Daily labor/merchant.

^c^ Variables with *p* ≥ 0.25 in bivariable analysis were not included in the multivariable model.

## 4 Discussions

In this study, the prevalence of the short birth interval was 29.20% (95% CI: 26.63, 31.90%). This finding was consistent with studies conducted in Iran (28.5%) [28] and Dabat district (30.60%) [29]. This finding was much lower than findings from Uganda (52.4%) [30] and northwest Ethiopia (43.4%) [18] and higher than findings from rural India (13%) [31]. The variation observed could be due to the difference in the sample size used, and socio‐demographic, study setting, behavioral, and cultural factors differ, resulting in significant variation. Despite the relatively lower prevalence observed, short birth interval remains a public health concern. Furthermore, the EDHS 2024–25 reports a relatively high total fertility rate (4.0 children per woman), particularly in rural areas, underscoring the need to strengthen family planning utilization and promote optimal birth spacing [14].

This study showed that women in rural area settings were more likely to have short birth intervals compared with women in urban area settings, which is consistent with previous studies conducted in the sub‐Sahara Africa and developing region of Ethiopia [32, 33]. This could be associated with poor awareness of optimal birth intervals. This outcome may be attributable to enhanced social services and increased access to knowledge, possibilities for education, and work in urban areas than in rural areas [34].

The household wealth index was found to be significantly associated with short birth interval. Women in rich households were less likely to have a short birth interval compared with women in poor households. This finding is similar to other studies performed in the Arbaminch district and the southern part of Ethiopia [10, 16]. This may be due to the fact that mothers from rich households had enough money and had greater access to health care information, such as postnatal consultations about healthy timing and spacing of pregnancy, family planning techniques for keeping adequate birth interval, and affordable health care services and materials [29].

In addition, this study showed that women who use contraception before their last pregnancy were less likely to have short birth interval as compared with women who did not use contraception. This was in line with prior studies conducted in Arbaminch district [10], Dembecha district [19], and North Wollo, Amhara, Ethiopia [7]. This could be the use of contraception can reduce and prevent the chances of unwanted pregnancy, lowering fertility and lengthening the birth interval [35].

Similarly, mothers who breastfed their preceding child for 24 and above months or more were less likely to have a short birth interval than mothers who breastfed their preceding child for less than 12 months. This finding is similar to studies done in Ethiopia [13, 36]. This might be due to the fact that breastfeeding extends the period of the interbirth interval through negative hormonal feedback. Breastfeeding leads to the secretion of prolactin hormone from the pituitary gland and lower follicular secretion hormone and luteinizing hormone levels in the blood. Subsequently, ovulation is delayed and the amenorrhea period prolongs, reducing the risk of fertility [37]. This study also identified husband/partner education as an important determinant of short birth interval. It revealed that mothers whose husbands had a primary education were 0.542 times less likely to have a short birth interval than those with an illiterate husband. This finding was consistent with studies done in Ethiopia and the Dembecha district [6, 19]. This might be the fact that educated husbands were more likely to cooperate with their wives in using family planning methods and value the importance of birth spacing [34].

Furthermore, the age of mothers at first marriage had a statistically significant association with short birth interval. This study revealed that women who married at the age of 18 and above were 0.611 times less likely to have short birth interval than women who married at an age below 18 years. This finding is in agreement with evidence generated from Bangladesh [38] and Nepal [39]. The reason might be that women who married at the age of 18 years and above would have a better opportunity for getting information and education. Besides, having more power in making decisions on future fertility and growing older might have resulted in a decline in women's fecundity, which increases the time it takes to become pregnant [19].

Moreover, women who have a desire to have six or more children had 2.048 times greater risk of short birth interval compared to those with a desire for fewer than six children. This finding is consistent with a studies conducted in Northern Ethiopia [4], rural developing communities of Southern Ethiopia [40], and Kassala, Eastern Sudan [41]. This might be due to the fact that parents and their community members have a desire for more children because of socio‐cultural and religious interests, in which the use of modern contraceptives for child spacing has not been practiced yet [42]. Furthermore, the lifestyle of the community is purely dependent on livestock, in which having more children is considered advantageous to get more keepers for their cattle. Thus, this perception of the community towards more children has been one of the contributors to short birth interval in these study areas of the country.

### 4.1 Strength and Limitation of the Study

The study's strength is that it used a community‐based study design to evaluate the predictors of short birth intervals in community, and the analysis uses primary data and assures the quality of data with the standardized data collection tool, and pretest was done before the actual data collection. As a limitation, the study was a cross‐sectional study design, which may not establish a temporal relationship between cause and effect. The 5‐year retrospective recall of reproductive events may have introduced recall bias, potentially leading to underestimation or overestimation of short birth intervals. There might be a possibility of recall, limit social desirability, and interviewer bias due to self‐reported data. Additionally, the findings are specific to the Awi zone, and caution should be taken when generalizing results to other regions with different socio‐demographic and cultural contexts. However, attention was given to the study procedures, including the process of training data collectors and close supervision throughout the activity to minimize the expected biases.

## 5 Conclusions

In Awi zone, the prevalence of short birth interval is still high. Husband/parent education level, use of contraceptive before last pregnancy, place of residence, wealth index of household, mother age at first marriage, duration of breastfeeding, and ideal number of children were statistically associated with a short birth interval. Therefore, policy makers and stakeholders strengthening family planning services, promoting optimal breastfeeding practices, delaying early marriage, and improving women's education and socioeconomic status are essential to encourage optimum birth interval. Furthermore, targeted community‐based interventions, particularly in rural areas, are recommended to reduce short birth intervals.

## Author Contributions

**Nigussie Adam Birhan:** conceptualization, investigation, writing – original draft, methodology, validation, visualization, writing – review and editing, software, formal analysis, data curation, supervision. **Kefale Tilahun Getahun:** investigation, writing – original draft, methodology, writing – review and editing. **Zelalem Meraf Wolde:** methodology, validation, software, formal analysis, supervision, data curation. **Kassahun Animut:** writing – original draft, writing – review and editing. **Denekew Bitew Belay:** writing – review and editing. **Yegnanew A. Shiferaw:** writing – review and editing.

## Ethics Statement

The study was conducted according to the Helsinki Declaration for Human Subjects Research. Ethical approval was obtained from the research and community service ethics review committee of the College of Natural and Computational Science, Injibara University, with a reference number of CNCS/PG/R/CS/V/Dean/255/23. Supportive letter of permission to conduct the study was obtained from the administrative office of each respective office. All study participants had verbal and written informed consent to confirm willingness to participate after explaining the objective of the study. Due to the low literacy level of some participants, the Ethics Review Committee approved the use of verbal consent. For those not willing to take part in the study, their right was respected to withdraw from the study. No personal identifiers were used on the data collection form. The recorded data was not accessed by a third person, and was kept confidentially and anonymously.

## Conflicts of Interest

The authors declare no conflicts of interest.

## Transparency Statement

The lead author, Nigussie Adam Birhan, affirms that this manuscript is an honest, accurate, and transparent account of the study being reported; that no important aspects of the study have been omitted; and that any discrepancies from the study as planned (and, if relevant, registered) have been explained.

## Data Availability

The data used to support the study findings are available from the corresponding author upon request.
